# Understanding the Prevalence of Medial Arterial Calcification Among Complex Reconstructive Patients: Insights from a Decade of Experience at a Tertiary Limb Salvage Center

**DOI:** 10.3390/jcm14020596

**Published:** 2025-01-17

**Authors:** Rachel N. Rohrich, Karen R. Li, Nicole C. Episalla, Khaleel Atkinson, Ryan P. Lin, Sami Ferdousian, Richard C. Youn, Karen K. Evans, Cameron M. Akbari, Christopher E. Attinger

**Affiliations:** 1 Department of Plastic and Reconstructive Surgery, MedStar Georgetown University Hospital, 3800 Reservoir Road, NW, Washington, DC 20007, USA; rachel.rohrich@medstar.net (R.N.R.); sf939@georgetown.edu (S.F.);; 2 Georgetown University School of Medicine, Washington, DC 20007, USA; 3 Department of Vascular Surgery, MedStar Georgetown University Hospital, Washington, DC 20007, USA

**Keywords:** medial arterial calcification, limb salvage, free tissue transfer, local flap, lower extremity amputation, peripheral arterial disease

## Abstract

**Background**: Medial arterial calcification (MAC), a distinct form of vascular pathology frequently coexisting with peripheral arterial disease (PAD), poses unique challenges in limb salvage among patients with diabetes, chronic kidney disease, and end-stage renal disease. This study examines the incidence of MAC and its impact on limb salvage outcomes over a decade of experience at a tertiary limb salvage center. **Methods**: A retrospective review of all complex lower extremity (LE) reconstructions using local flap (LF) or free tissue transfer (FTT), performed from July 2011 to September 2022, was conducted. Patients were classified into MAC and No MAC groups based on pedal radiography evaluations using the Ferraresi MAC scoring system. The primary outcomes were major lower extremity amputation (MLEA), the need for postoperative vascular intervention, major adverse limb events (MALE; defined as the composite of any unplanned reoperation, MLEA, or postoperative revascularization attempt), and mortality. **Results**: During the study period, a total of 430 LE reconstructions were performed with LF or FTT. A total of 323 cases (75.1%) demonstrated no MAC while the remaining 107 (24.9%) demonstrated MAC. The MAC group exhibited significantly higher rates of diabetes, PAD, and renal disease. With a follow-up duration of 17.0 (IQR: 33.9) months, the MAC group demonstrated a significantly higher rate of MLEA (24.3% vs. 13.0%, *p* = 0.006), postoperative vascular intervention (23.4% vs. 8.7%, *p* < 0.001), MALE (57.0% vs. 25.7%, *p* < 0.001), and mortality (28.0% vs. 9.9%, *p* < 0.001). Multivariate analysis identified MAC as independently predictive of MALE (OR: 1.8, CI: 1.1–3.0, *p* = 0.033). **Conclusion**: MAC is prevalent among surgical candidates for limb salvage. Patients with MAC represent a significant medical and reconstructive challenge. Radiographic screening for MAC should be considered in all limb salvage candidates with LE wounds, especially in those with diabetes and kidney disease. Assessing MAC is important for better evaluating risk factors and surgical options so as to optimize outcomes in this challenging population.

## 1. Introduction

Peripheral arterial disease (PAD) currently affects over 200 million people globally and is becoming more prevalent in an aging world population [1,2]. Concomitantly, the proportion of individuals with conditions that exacerbate vascular disease and calcification—diabetes mellitus (DM) and chronic kidney disease (CKD)—is also rising [3,4,5,6]. These diseases, independently or combined, often affect the vascularity of the lower extremity (LE) resulting in ischemia, and ultimately, wounds requiring significant reconstruction or amputation. Reconstruction, however, is often challenging, namely due to the patient comorbidity burden that results in damage to the arterial vessels supplying the LE. Medial arterial calcification (MAC) is an underdiagnosed product of these systemic medical conditions that affects the arterial walls of the LE [7,8]. MAC is a chronic condition characterized by calcium phosphate deposition in the medial layer of the arterial wall that leads to arterial stiffening and reduced compliance. It is a pathology that is separate and distinct from PAD and is strongly associated with cardiovascular events and death in aging patients with DM, CKD, and end-stage renal disease (ESRD) [7,9,10,11,12,13,14].

Due to our lack of understanding of MAC’s pathogenesis, there is currently no standard causal treatment for MAC [15]. However, there may be potential in investigational anti-calcification therapeutics and other pharmacologic interventions targeting various stages of the calcification process [16,17,18]. Ongoing omics approaches to MAC research (e.g., proteomics, metabolomics, single-cell transcriptomics) are contributing to a better understanding of the disease process, possible future treatment, and prevention targets [9,19,20,21]. In this context, patients with MAC are likely to present with ischemic LE wounds that require the expertise of plastic and reconstructive surgery teams. It is therefore important for these teams to have a comprehensive understanding of this patient population.

Indeed, in patients with LE ischemia, MAC has shown to be an independent prognostic factor in predicting major LE amputation, as it is frequently found in distal arterial beds of limbs amputated due to ischemia [22,23,24,25]. The literature has coined MAC a “silent killer of the leg”, which, interestingly, has received little attention from reconstructive surgery teams who are often responsible for treating patients with ischemic LE wounds complicated by MAC [8]. Our goal as a tertiary limb salvage center with years of experience in treating patients with MAC is to draw attention to this major contributor to LE wounds and its impact on reconstructive outcomes.

Primary reconstructive options (beyond conservative management and skin grafting) at our institution for limb salvage in this population include local flaps (LF) and free tissue transfer (FTT). Although these are plastic surgery techniques, their success depends on coordinated care with a vascular surgery team. We have therefore adopted a “vasculo-plastic” approach at our institution, which has helped us expand the typical criteria of “reconstructable” limb salvage patients and improve limb salvage rates [26,27]. While previous studies have reported limb salvage outcomes in patients with MAC treated with endovascular interventions and conservative wound management approaches (e.g., debridement, negative pressure wound therapy, and traditional dressing care), none have explored the outcomes of more advanced reconstructive interventions combined with vascular optimization in this population [28]. Thus, this study examines the incidence of MAC among patients undergoing high-level limb salvage procedures at our institution and presents outcomes from our vasculo-plastic approach in the severely disadvantaged population with both MAC and PAD to contribute to the growing body of literature on MAC and limb salvage.

## 2. Methods

### 2.1. Study Design and Variable Definitions

After institutional board approval, a retrospective chart review of all patients receiving LE reconstruction with LF or FTT from July 2011 to September 2022 at a single institution was conducted. All LFs were performed by the senior author (C.E.A) and all FTTs were performed by a single senior microsurgeon (K.K.E). All patients underwent diagnostic or therapeutic angiograms, which were performed by a single senior vascular surgeon (C.M.A.).

Electronic medical records were reviewed to collect patient demographics, wound characteristics, vascular details, laboratory values, intraoperative information, and postoperative outcomes. The Charlson Comorbidity Index (CCI), a validated tool that assesses 10-year mortality, was used to quantify the comorbidity burden of each patient [29].

The vascularity of the extremity was characterized by the patency of the anterior tibial (AT), posterior tibial (PT), and peroneal arteries and further categorized into patent, occluded, or occluded with distal reconstitution. Each patient was given a vessel runoff (VRO) score, which was the sum of patent arteries providing in-line flow to the foot. VRO was between zero to three. If a patient required intervention, the type and location were recorded.

Immediate and long-term outcomes were collected. Immediate flap success was defined as flap viability without need for revision surgery between postoperative days (PODs) 0 and 12. Other immediate outcomes assessed included return to the operating room due to concerns over flap viability between PODs 0 and 7 and partial flap necrosis from PODs 0 to 12. Long-term outcomes included the rates of ipsilateral major lower extremity amputation (MLEA; a below-knee amputation [BKA] or proximal), the need for postoperative vascular intervention, ambulatory status at final follow-up, major adverse limb events (MALE; defined as any reoperation, MLEA, or postoperative revascularization attempt), and mortality. Mortality was defined as documented evidence of death in the patient’s medical record or an online obituary. Mortality rates were recorded as overall mortality, and at 1, 2, and 3 years postoperatively. Primary outcomes for this study were the rates of postoperative revascularization, MLEA, MALE, and mortality.

### 2.2. Assessment of MAC and Study Groups

**Figure 1:**
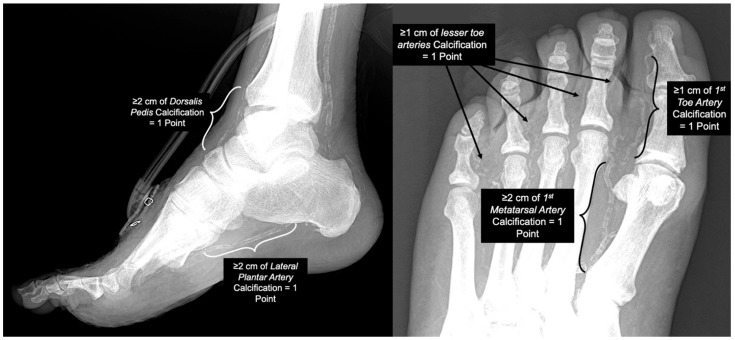
The five vascular sites assessed in the Ferraresi pedal MAC scoring systems utilized in this study.

Preoperative pedal radiographs were used to determine the presence of pedal MAC according to the scoring system proposed by Ferraresi et al. in their 2021 publication. The scoring system is based on the extent of calcification seen within predefined vascular sites (Figure 1). Two independent reviewers scored each radiograph. A senior vascular surgeon settled scoring conflicts. All observers underwent a standardized training process. For this study, patients were classified into two groups as follows: No MAC (Ferraresi pedal MAC score of 0–1) and MAC (Ferraresi pedal MAC score of 2–5) [28]. Interrater reliability was assessed using Cohen’s Kappa statistic. The following cut-offs were used for the interpretation: ≤0 as no agreement, 0.01–0.20 as slight agreement, 0.21–0.40 as fair agreement, 0.41–0.60 as moderate agreement, 0.61–0.80 as substantial agreement, and 0.81–1.00 as almost perfect agreement [30].

### 2.3. Vasculo-Plastic Perioperative Protocol

#### 2.3.1. Preoperative Protocol for Vascular Optimization

All patients underwent arteriography prior to reconstruction as part of our preoperative algorithm to guide LF or FTT planning. Digital subtraction arteriography was performed under moderate sedation and approximately 12 cc of radiopaque contrast dye was used for each arteriogram. Significant stenoses or occlusions were treated with balloon angioplasty with adjunct stenting when necessary. Some patients required vascular reconstruction with a bypass.

#### 2.3.2. Flap Selection

Preoperatively, patients are considered for either LF or FTT based on overall health status, LE vasculature, wound characteristics, functional prognosis, and their ability to tolerate general anesthesia for a prolonged operation. At our institution, local flaps are the preferred first option and are utilized whenever feasible due to their shorter operative duration, reduced surgical morbidity, and better contour for functional shoe wear. Local flaps are indicated for smaller defects that can be adequately covered with pedicled or local tissue, particularly when the local vasculature is sufficient to support the flap. A description of local flap techniques and further indications have been previously outlined in detail by the senior author (C.E.A.) [31,32,33,34,35]. Free flaps are indicated in patients with larger defects with inadequate local vascularized and healthy tissue. Further FTT techniques, considerations, and indications at our institution have been described by the senior microsurgeon author (K.K.E.) in past publications [26,27,36,37]. Both options are complex and warrant careful surgical planning and patient optimization, including the confirmation of vascular status via arteriography [34,38]. For all patients, serial debridements were performed until healthy tissue and/or negative cultures were achieved prior to definitive reconstruction.

#### 2.3.3. Postoperative Vasculo-Plastic Surveillance

In the immediate postoperative setting (typically PODs 0–5), patients were closely monitored for signs of compromised flap perfusion, flap necrosis, and venous congestion. This was achieved by clinical examination and supplemented by perfusion monitoring tools (specifically in FTT) such as the Cook–Swartz Doppler (Cook Medical, Bloomington, IN, USA) and ViOptix tissue oximeter (ViOptix Inc., Newark, CA, USA) by the surgical team and trained nurses. If there were concerns over flap perfusion, patients underwent immediate operative exploration of the vasculature supplying the LF or FTT as well as a repeat diagnostic or therapeutic angiogram.

In the long term, patients were monitored very closely in our multidisciplinary wound clinic that consists of a core group of vascular, plastic, and podiatric surgeons, with support from the infectious disease, internal medicine, rheumatology, general surgery, hyperbaric medicine, and orthotist/prosthetist teams. The vascular surgery team is consistently available to assess LE perfusion at each clinic visit with handheld Doppler exams. Our team maintains a low threshold for repeat angiography to enable timely intervention if necessary. Clinical indicators such as the development of new ulcerations prompt re-assessment of vascular patency, including the patency of prior interventions.

### 2.4. Statistical Analysis

The following two groups were stratified based on MAC score: MAC and No MAC. Descriptive statistics were calculated for all patient data. Categorical variables were reported as counts and percentages. Normally distributed continuous variables were reported as the means and standard deviations; non-normally distributed continuous variables were represented as medians and interquartile ranges (IQR). To investigate differences between groups based on MAC, chi-square or Fischer’s exact tests were used for categorical variables and Student’s t-test or Mann–Whitney U tests for continuous variables. Multivariate linear regression analyses controlling for clinically significant covariates relevant to both MAC and our primary outcomes (DM, PAD, CKD, age, and reconstruction type) were conducted. Statistical analysis was performed using StataMP Software (StataCorp, LLC, College Station, TX, USA).

## 3. Results

### 3.1. Patient Characteristics

During the 10-year study period, a total of 430 LE reconstructions were performed with LF or FTT. A total of 323 cases (75.1%) demonstrated no MAC while the remaining 107 (24.9%) demonstrated MAC. The Cohen’s Kappa statistic was 0.733, indicating a substantial agreement between radiograph reviewers. Patient characteristics are shown in Table 1. Median age (61, IQR: 14 vs. 58, IQR: 19 years; *p* = 0.049) and BMI (31.5, IQR: 7.6 vs. 28.3, IQR: 8.0 kg/m^2^; *p* = 0.002) were significantly higher in the MAC group. The MAC group also demonstrated a significantly higher median CCI (6, IQR: 2 vs. 4, IQR: 3; *p* < 0.001), reflecting a 0% estimated 10-year survival rate among MAC patients. The MAC group demonstrated significantly higher rates of DM (93.5% vs. 52.9%, *p* < 0.001), PAD (82.2% vs. 47.7%, *p* < 0.001), CKD (24.3% vs. 12.7%, *p* = 0.004), ESRD (25.2% vs. 2.5%, *p* < 0.001), peripheral neuropathy (87.9% vs. 46.8%, *p* < 0.001), congestive heart failure (22.4% vs. 7.7%, *p* < 0.001), history of myocardial infarction (12.1% vs. 4.3%, *p* = 0.004), and previous transplant history (10.3% vs. 0.9%, *p* < 0.001) compared to the no MAC group. Notably, nearly half of MAC patients had some form of renal disease (49.5%) compared to 15.2% in the no MAC group (*p* < 0.001). Additionally, the MAC group demonstrated significantly higher preoperative values for ESR (*p* < 0.001), CRP (*p* < 0.001) and A1c (*p* < 0.001), and significantly lower values of albumin (*p* < 0.001) and prealbumin (*p* < 0.001). Patients had a median wound size of 55.5 (IQR: 64) cm^2^, which did not differ between MAC groups (*p* = 0.993; Table 2). Wound location within the LE did not differ between groups, with most wounds located on the foot (49.1%) or ankle (34.5%).

### 3.2. Preoperative Vascular Status

The preoperative vascular characteristics of those who underwent a preoperative angiogram (n = 347) are summarized in Table 3. The MAC group demonstrated significantly higher rates of occlusion and lower rates of patency of all three vessels (*p* < 0.001 for all), in addition to a lower VRO (*p* < 0.001). A significantly higher proportion of patients in the MAC group required endovascular intervention (41.1% vs. 14.6%, *p* < 0.001) prior to reconstruction. Most interventions were balloon angioplasty (98.9%), with four cases (4.4%) requiring bypass during their hospital stay.

### 3.3. Operative Details

Operative details are summarized in Table 4. The median operative duration was 347 (IQR: 239) minutes. A significantly higher proportion (*p* < 0.001) of MAC patients received LF reconstruction (66.4% vs. 30.3%) compared to the no MAC group that more commonly received FTT (69.7% vs. 30.3%).

### 3.4. Clinical Outcomes

Clinical outcomes are summarized in Table 5. A significantly higher proportion of MAC patients experienced flap complications (56% vs. 31.0%, *p* < 0.001) compared to no MAC patients, particularly flap necrosis (5.6% vs. 1.9%, *p* = 0.041), dehiscence (30.8% vs. 18.0%, *p* = 0.005), and infection (46.7% vs. 17.3%, *p* < 0.001). Immediate flap success, which occurred in 418 cases (97.2%), did not differ by group (*p* = 0.183). With a follow-up duration of 17.0 months (IQR: 33.9), the MAC group demonstrated a significantly higher rate of MLEA (24.3% vs. 13.0%, *p* = 0.006) and postoperative vascular intervention (23.4% vs. 8.7%, *p* < 0.001). MAC patients required MLEA significantly earlier following reconstruction, with a median postoperative time to amputation of 69 days (IQR: 184) compared to 270 days (IQR: 362) in non-MAC patients (*p* < 0.001). MALE occurred at a significantly higher rate in the MAC group, affecting more than half of patients with MAC (61.7% vs. 40.9%, *p* < 0.001). MAC patients were also less likely to be ambulatory at their final follow-up (72.6% vs. 85.7%, *p* = 0.001).

Overall mortality was significantly higher in the MAC group (29.9% vs. 10.3%, *p* < 0.001). While 1-year mortality showed a higher trend in MAC patients (6.5% vs. 2.8%, *p* = 0.075), it was not statistically significant. However, 2-year (12.2% vs. 5.3%, *p* = 0.015) and 3-year mortality (14.0% vs. 5.6%, *p* = 0.004) were significantly higher in the MAC group. Among MAC patients that experienced mortality within the study period, rates of DM (100.0%), neuropathy (90.6%), PAD (81.3%), MLEA (28.1%), congestive heart failure (28.1%), CKD (28.1%), and ESRD (28.1%) were prevalent. At the time of reconstruction, the average age among these patients was 62.6 ± 9.6 years.

Lastly, a sub-analysis comparing patients who underwent postoperative MLEA to those that did not demonstrated that those with MLEA had a significantly lower ambulatory rate (56.1% vs. 87.3%, *p* < 0.001) and a higher overall mortality rate (27.9% vs. 12.7%, *p* = 0.001).

### 3.5. Multivariate Analysis

In multivariate analyses controlling for clinically relevant co-variates, MAC remained significantly and independently predictive only of MALE (odds ratio [OR]: 1.8; confidence interval [CI]: 1.0–2.9, *p* = 0.043) and a shorter time to postoperative MLEA (β: −309.7; CI: −565.8, −53.5, *p* = 0.019), but not of mortality, postoperative revascularization, or MLEA. Further results from multivariate analyses are shown in Table 6, Table 7, Table 8, Table 9, Table 10 and Table 11.

## 4. Discussion

In this large patient population with chronic LE wounds requiring complex surgical reconstruction, we have demonstrated that nearly 25% were affected by MAC, 88.2% of which were also affected by PAD. In line with the current literature on MAC, patients with MAC in our study were older and demonstrated a significantly higher comorbidity burden, with nearly all MAC patients having DM with peripheral neuropathy and almost half having renal disease. Additionally, MAC patients demonstrated a significantly higher BMI, which is known to affect wound healing and postoperative outcomes [39,40,41]. Patients with MAC had significantly higher rates of MLEA and MALE, and more often required a postoperative vascular intervention. Importantly, in line with other studies, a significantly higher mortality rate (nearly 30%) was observed in the MAC group during their 21.7-month follow-up period, which is likely related to the high incidence of chronic systemic disease in this cohort [42,43,44]. The presence of MAC may therefore serve as a predictor of and warning sign for poor outcomes in patients requiring reconstruction for chronic LE wounds. Though MAC remained predictive of only MALE and shorter time to MLEA based on multivariate analysis, it is important to recall that patients with MAC are highly comorbid individuals with complex disease processes that dramatically increase their risk for overall morbidity and mortality. Therefore, the presence of MAC should prompt physicians to optimize these patients in the face of an increased risk of both poor operative outcomes as well as morbidity and mortality.

MAC, a small artery disease (SAD), represents a distinct pathological process from the atherosclerotic changes typical of “big artery disease” (BAD) [7,28,45]. Unlike stenotic plaques forming in the intimal layer in BAD, MAC leads to calcification within the medial layer of the vessel wall, resulting in arterial stiffening and reduced compliance [9,46]. This reduces distal perfusion pressure, which may not be evident in a traditional arterial exam, further complicating the vascular assessment in PAD patients with concomitant MAC. While patients with BAD benefit from endovascular revascularization techniques (e.g., balloon angioplasty), there is currently no standard therapeutic treatment for MAC; management currently focuses on lifestyle modifications, medical therapy, and glycemic control [9]. Because MAC is characterized by diffuse calcification along the arterial wall, affecting small vessels, it is less responsive to such revascularization techniques [9]. Thus, for patients with both MAC and PAD, optimizing distal perfusion becomes increasingly important in supporting limb salvage efforts. Given that MAC’s presence in the pedal arteries signals a broader systemic vulnerability to vascular and metabolic dysfunction, early recognition through simple radiographic imaging can guide clinicians in identifying high-risk patients to prioritize care that maximizes the potential for limb preservation [47,48,49].

The high incidence of MAC in our study’s limb salvage population highlights the need for the clinical recognition of MAC alongside traditional PAD in patients with chronic LE wounds. The simplicity of identifying MAC through basic pedal X-rays makes this possible, even in resource-limited settings. Ferraresi’s MAC score, which can be determined by accessible and cost-effective X-rays, is quick and easy to obtain and relatively inexpensive. Our study demonstrates that, despite advances in reconstructive interventions and preoperative optimization efforts, MAC remains an important predictor of poor limb outcomes. We therefore recommend screening for MAC in comorbid patients with LE wounds by obtaining pedal X-rays with the goal of optimizing these patients for improved wound healing and overall survival. Further, as MAC is known to run a prolonged and clinically silent course, the use of this scoring system in other settings where patients likely to have MAC are already engaged in the healthcare system (e.g., diabetic foot clinics, dialysis centers, endocrinology offices, and trauma units) would enable the broader identification of at risk-individuals. Identifying these patients before they reach end-stage limb salvage could allow for the earlier contact with vascular specialists and the appropriate lifestyle modifications aimed at mitigating complex surgical interventions.

Of particular significance is that MAC patients in our cohort demonstrated a mortality rate of nearly 30%, compared to roughly 10% in non-MAC patients. This finding must be interpreted in the context of the poor comorbidity profile of the MAC group. Notably, all MAC patients who passed away within the study period had one or more conditions associated with metabolic syndrome as follows: 100% had DM, 81.3% had PAD, 56.3% had kidney disease, 12.5% had a history of myocardial infarction, 28.1% had congestive heart failure suggesting coronary disease, 6.3% had liver disease, and the mean BMI was 32.6. These findings suggest that MAC may play a role in metabolic syndrome, which may cumulatively drive poor outcomes [42,43,48,49,50,51,52,53]. Thus, decisions regarding limb salvage versus amputation in these patients should be approached with caution, as the high composite risk for mortality and/or amputation may contraindicate aggressive interventions with long operative durations and hospital stays in favor of a more conservative approach that may still produce a functional outcome [54,55]. However, without a comparative analysis to a population that underwent primary amputation, such conclusions about limb salvage decisions cannot be made. Conversely, it is also important to note that there exists a subset of MAC patients who achieve successful outcomes and limb salvage. Future research should aim to identify these protective factors to further guide treatment.

Overall, we believe that a coordinated, multidisciplinary approach is essential for managing any limb salvage patient—but especially patients with MAC who harbor chronic medical conditions, particularly DM and renal disease. Efforts to control blood glucose levels, manage renal disease effectively, and address broader cardiovascular risk factors should be a priority in MAC patients when detected early, as medical management could mitigate the progression of MAC and potentially reduce the need for major limb reconstruction, reduce healthcare costs, and improve overall limb salvage outcomes [41]. Our results call for integrated care structures that not only improve outcomes for the ischemic limb but also prioritize systemic health optimization to prevent severe complications that compromise limb viability and survival.

### Limitations

Our results must be considered in the context of this study’s limitations, many of which stem from its retrospective design wherein the results rely on both the accuracy of documentation in clinical records and collection by chart reviewers. Mortality data were obtained from patient charts and online obituaries, which may introduce potential inaccuracies or may represent an incomplete capture of all deaths during the study period. Secondly, MAC scoring was based on an X-ray review, which may have introduced some inaccuracies in score assignment; however, this system has been shown to be highly reproducible and consistent among reviewers [28]. Additionally, patients without available radiographs or with amputations precluding a full score assignment (i.e., transmetatarsal amputation) were excluded from the analysis, which may have introduced selection bias to our findings. Because we did not have preoperative arteriography data for all patients, we were unable to include a measure of PAD severity (i.e., vessel runoff) in our multivariate analyses without omitting a significant portion of our study population (nearly 20%). The severity of PAD likely impacts outcomes and should be considered in future analyses. It is also important to note that all LE reconstructions were performed by two highly skilled and experienced surgeons in complex limb salvage, in collaboration with a talented vascular surgeon specialized in managing this patient population, which may limit the generalizability of our relatively favorable results in patients with severe MAC. Lastly, we included reconstructive patients who underwent both LF and FTT, potentially introducing variability in outcomes related to the reconstructive type. Our study’s goal was not to compare these reconstructive methods, but rather to evaluate long-term outcomes and characterize the presence of MAC in patients requiring higher-level reconstruction. However, we controlled for any confounding effects of the reconstructive type in our multivariate analyses.

## 5. Conclusions

Patients with severe MAC present a significant clinical challenge for all teams involved in their care. Our findings demonstrate the need to initiate a broader conversation on MAC within vascular, internal medicine, and reconstructive surgery literature and practice. Incorporating MAC evaluation into the clinical examination of patients presenting with PAD or LE ulceration in its early stages may be helpful, particularly in patients with chronic conditions strongly associated with MAC progression (CKD and DM). These patients necessitate a collaborative approach led by both plastic and vascular surgery—the vasculo-plastic model—to make educated limb salvage decisions that prioritize the option with the highest likelihood for success guided by both vascular and soft tissue viability, patient comorbidities, risk of mortality, and functional prognosis.

## Figures and Tables

**Table 1:** Patient characteristics.

	All Limbs	No MAC	MAC	*p*-Value
	No. (%)	No. (%)	No. (%)	
Demographics	430 (100.0)	323 (75.1)	107 (24.9)	
Age, years (median [IQR])	59 [17]	58 [19]	61 [14]	0.049 *
BMI, kg/m^2^ (median [IQR])	29.1 [8.6]	28.3 [8.0]	31.5 [7.6]	0.002 *
Hospital LOS, days (median [IQR])	22 [17]	22 [16]	22 [18]	0.966
Postoperative LOS, days (median [IQR])	11 [9]	11 [8]	10 [10]	0.110
Smoking History				0.002 *
Never	276 (64.2)	198 (61.3)	78 (72.9)	
Past	100 (23.3)	75 (23.2)	25 (23.4)	
Current	54 (12.6)	50 (15.5)	4 (3.7)	
**Comorbidities**				
CCI (median [IQR])	5 [3]	4 [3]	6 [2]	<0.001 *
Diabetes	271 (63.0)	171 (52.9)	100 (93.5)	<0.001 *
Peripheral vascular disease	242 (56.3)	154 (47.7)	88 (82.2)	<0.001 *
End-stage renal disease	35 (8.1)	8 (2.5)	27 (25.2)	<0.001 *
Chronic kidney disease	67 (15.6)	41 (12.7)	26 (24.3)	0.004 *
Any kidney disease	102 (23.7)	49 (15.2)	53 (49.5)	<0.001 *
Liver disease	17 (4.0)	10 (3.1)	7 (6.5)	0.113
Peripheral neuropathy	245 (57.0)	151 (46.8)	94 (87.9)	<0.001 *
Congestive heart failure	49 (11.4)	25 (7.7)	24 (22.4)	<0.001 *
COPD	15 (3.5)	11 (3.4)	4 (3.7)	0.871
History of CVA/TIA	32 (7.4)	20 (6.2)	12 (11.2)	0.086
History of MI	27 (6.3)	14 (4.3)	13 (12.2)	0.004 *
History of transplant	14 (3.3)	3 (0.9)	11 (10.3)	<0.001*
**Preoperative Labs**				
WBC, cells/µL (median [IQR])	7.4 [3.2]	7.4 [3.1]	8.0 [4.5]	0.083
ESR, mm/h (median [IQR])	64 [67]	55.5 [63.0]	93 [71]	<0.001 *
CRP, mg/L (median [IQR])	18.5 [45.1]	16.2 [38.3]	34 [76.3]	<0.001 *
A1c, % (median [IQR])	6.7 [2.6]	6.4 [2.3]	7.5 [3.2]	<0.001 *
Albumin, g/dL (median [IQR])	3.1 [1.0]	3.1 [1.0]	2.8 [1.0]	<0.001 *
Prealbumin, mg/dL (median [IQR])	18.1 [9.8]	18.8 [8.8]	14.5 [9.0]	<0.001 *
Hemoglobin, g/dL (median [IQR])	9.6 [2.3]	9.8 [2.5]	9.2 [2.0]	<0.001 *
Platelet, cells/µL (median [IQR])	265 [129]	264 [117]	273 [166]	0.849
Hypercoagulability traits	258 (60.0)	200 (61.9)	58 (54.2)	0.158

* Values in bold indicate statistical significance, *p* < 0.05. Abbreviations: MAC, medial arterial calcification; IQR, interquartile range; BMI, body mass index; LOS, length of stay; CCI, Charlson Comorbidity Index; WBC, white blood cells; ESR, erythrocyte sedimentation rate; CRP, C-reactive protein; A1c, glycated hemoglobin; g/dL, grams per deciliter; mg/dL, milligrams per deciliter; µL, microliters; mm/h, millimeters per hour; CVA, cerebrovascular accident; TIA, transient ischemic attack; MI, myocardial infarction; COPD, chronic obstructive pulmonary disease.

**Table 2:** Wound characteristics.

	All Limbs	No MAC	MAC	*p*-Value
	No. (%)	No. (%)	No. (%)	
	430 (100.0)	323 (75.1)	107 (24.9)	
Wound area, cm^2^ (median [IQR])	55.5 [64]	56 [68]	54 [52]	0.993
Wound location				0.401
Dorsal Foot	32 (12.3)	22 (12.4)	10 (12.1)	
Plantar Foot	47 (18.0)	31 (17.4)	16 (19.3)	
Medial or Lateral Foot	49 (18.8)	30 (16.9)	19 (22.9)	
Ankle	90 (34.5)	68 (38.2)	22 (26.5)	
Lower Leg	43 (16.5)	27 (15.2)	16 (19.3)	
Charcot arthropathy	63 (14.7)	43 (13.4)	20 (18.7)	0.177
Osteomyelitis on DOS	199 (46.4)	155 (48.1)	44 (41.1)	0.207

Abbreviations: MAC, medial arterial calcification; IQR, interquartile range; cm^2^, square centimeters; DOS, day of surgery.

**Table 3:** Preoperative vascular characteristics ^1^.

	All Limbs	No MAC	MAC	*p*-Value
	No. (%)	No. (%)	No. (%)	
Preoperative arteriogram	**347 (100.0)**	**259 (74.6)**	**88 (25.4)**	
**Arteriogram Findings**				
Anterior tibial artery				0.001 *
Patent	230 (66.7)	186 (72.4)	44 (50.6)	
Occluded	73 (21.2)	46 (17.9)	27 (31.0)	
Occluded with distal reconstitution	41 (11.9)	25 (9.7)	16 (18.4)	
Posterior tibial artery				<0.001 *
Patent	245 (71.2)	201 (78.2)	44 (50.6)	
Occluded	66 (19.2)	39 (15.2)	27 (31.0)	
Occluded with distal reconstitution	33 (9.5)	17 (6.6)	16 (18.4)	
Peroneal artery				<0.001 *
Patent	285 (82.6)	225 (86.9)	60 (69.8)	
Occluded	43 (12.5)	22 (8.5)	21 (24.4)	
Occluded with distal reconstitution	17 (4.9)	12 (4.6)	5 (5.8)	
Vessel runoff				<0.001 *
0	19 (5.6)	11 (4.3)	8 (9.4)	
1	58 (16.9)	30 (11.6)	28 (32.9)	
2	97 (28.3)	71 (27.5)	26 (30.6)	
3	169 (49.3)	146 (56.6)	23 (27.1)	
**Vascular Intervention**				
Required vascular intervention	91 (21.2)	47 (14.6)	44 (41.1)	<0.001 *
Intervention type				
Balloon angioplasty	90 (98.9)	46 (97.9)	44 (100.0)	0.331
With Stent	4 (4.4)	3 (6.4)	1 (2.3)	0.339
Bypass	4 (4.4)	2 (4.3)	2 (4.6)	0.946

^1^ Reported out of the total number of those that received an arteriogram (347 total: 259 No MAC, 88 MAC). * Values in bold indicate statistical significance, *p* < 0.05.

**Table 4:** Intraoperative details.

	All Limbs	No MAC	MAC	*p*-Value
	No. (%)	No. (%)	No. (%)	
	430 (100.0)	323 (75.1)	107 (24.9)	
Number of preoperative debridements (median [IQR])	2 [1]	2 [1]	2 [1]	0.608
Operative duration	347 [239]	368 [212]	218 [239]	<0.001 *
Reconstruction type				
Local flap	169 (39.3)	98 (30.3)	71 (66.4)	<0.001 *
Free tissue transfer	261 (60.7)	225 (69.7)	36 (33.6)	

* Values in bold indicate significance, *p* < 0.05. Abbreviations: MAC, medial arterial calcification; IQR, interquartile range.

**Table 5:** Clinical outcomes.

	All Limbs	No MAC	MAC	*p*-Value
	No. (%)	No. (%)	No. (%)	
	430 (100.0)	323 (75.1)	107 (24.9)	
**Flap Complications**				
Any flap complication	160 (37.2)	100 (31.0)	60 (56.1)	<0.001 *
Takeback (POD0-7)	13 (3.0)	9 (2.8)	4 (3.7)	0.618
Flap Salvage ^1^	9 (69.2)	6 (66.7)	3 (75.0)	1.000
Partial flap necrosis	12 (2.8)	6 (1.9)	6 (5.6)	0.041 *
Dehiscence	91 (21.2)	58 (18.0)	33 (30.8)	0.005 *
Flap hematoma	20 (4.7)	14 (4.3)	6 (5.6)	0.588
Infection	106 (24.7)	56 (17.3)	50 (46.7)	<0.001 *
Immediate flap success (POD0-12)	418 (97.2)	316 (97.8)	102 (95.3)	0.183
**Long-Term Follow-Up Outcomes**				
Final follow-up duration, months (median [IQR])	17.0 [33.9]	15.7 [30.2]	21.7 [36.9]	0.526
Postoperative vascular intervention	53 (12.4)	28 (8.7)	25 (23.4)	<0.001 *
Postoperative BKA	68 (15.8)	42 (13.0)	26 (24.3)	0.006 *
Time to amputation, days (median [IQR])	174 [339]	270 [362]	69 [184]	<0.001 *
MALE	198 (46.1)	132 (40.9)	66 (61.7)	<0.001 *
Ambulatory at final follow-up	347 (82.4)	270 (85.7)	77 (72.6)	0.001 *
**Mortality**				
Mortality during follow-up period	65 (15.2)	33 (10.3)	32 (29.9)	<0.001 *
Time to mortality, days (median [IQR]) ^2^	842 [1423]	553 [1495]	1142 [1345]	0.540
1-year mortality	16 (3.7)	9 (2.8)	7 (6.5)	0.075
2-year mortality	30 (7.0)	17 (5.3)	13 (12.2)	0.015 *
3-year mortality	33 (7.7)	18 (5.6)	15 (14.0)	0.004 *

* Values in bold indicate statistical significance, *p* < 0.05. ^1^ Flap salvage represented out of the total number of flaps that underwent takeback. ^2^ Time to mortality available for 60 patients only (29 No MAC, 31 MAC). Abbreviations: MAC, medial arterial calcification; IQR, interquartile range; POD, postoperative day; BKA, below-knee amputation; MALE, major adverse limb event.

**Table 6:** Multivariate model for major adverse limb events (MALE).

Co-Variate	OR (95% CI)	*p*-Value
MAC	1.7 (1.0, 2.9)	0.043 *
Diabetes mellitus	1.8 (1.2, 2.9)	0.010 *
PAD	1.9 (1.2, 2.9)	0.006 *
CKD	1.1 (0.6, 1.8)	0.858
Age	1.0 (0.8, 1.9)	0.094
Reconstruction type		
Local flap	1 [Reference]	-
Free tissue transfer	1.2 (0.2, 1.9)	0.380

* Values in bold indicate statistical significance. Abbreviations: OR, odds ratio; CI, confidence interval; MAC, medial arterial calcification; PAD, peripheral arterial disease; CKD, chronic kidney disease.

**Table 7:** Multivariate model for major lower extremity amputation (MLEA).

Co-Variate	OR (95% CI)	*p*-Value
MAC	1.8 (0.9, 3.4)	0.084
Diabetes mellitus	3.1 (1.5, 6.6)	0.003 *
PAD	1.1 (0.6, 2.1)	0.719
CKD	1.4 (0.7, 2.8)	0.361
Age	1.0 (0.9, 1.0)	0.004 *
Reconstruction type		
Local flap	1 [Reference]	-
Free tissue transfer	1.4 (0.7, 2.5)	0.311

* Values in bold indicate statistical significance. Abbreviations: OR, odds ratio; CI, confidence interval; MAC, medial arterial calcification; PAD, peripheral arterial disease; CKD, chronic kidney disease.

**Table 8:** Multivariate model for time to major lower extremity amputation (MLEA).

Co-Variate	β Coefficient (95% CI)	*p*-Value
MAC	−309.7 (−565.8, −53.5)	0.019 *
Diabetes mellitus	−158.6 (−494.6, 178.5)	0.351
PAD	−93.4 (−368.8, 182.0)	0.501
CKD	−6.7 (−292.5, 279.2)	0.963
Age	5.5 (−4.8, 15.8)	0.287
Reconstruction type		
Local flap	1 [Reference]	-
Free tissue transfer	−343.7 (−589.4, −97.9)	0.007 *

* Values in bold indicate statistical significance. Abbreviations: CI, confidence interval; MAC, medial arterial calcification; PAD, peripheral arterial disease; CKD, chronic kidney disease.

**Table 9:** Multivariate model for postoperative vascular intervention.

Co-Variate	OR (95% CI)	*p*-Value
MAC	1.5 (0.8, 3.0)	0.220
Diabetes mellitus	3.8 (1.4, 10.4)	0.009 *
PAD	5.6 (2.1, 14.9)	0.001 *
CKD	1.1 (0.5, 2.3)	0.792
Age	1.0 (1.0, 1.0)	0.191
Reconstruction type		
Local flap	1 [Reference]	-
Free tissue transfer	1.0 (0.5, 2.0)	0.962

* Values in bold indicate statistical significance. Abbreviations: OR, odds ratio; CI, confidence interval; MAC, medial arterial calcification; PAD, peripheral arterial disease; CKD, chronic kidney disease.

**Table 10:** Multivariate model for mortality.

Co-Variate	OR (95% CI)	*p*-Value
MAC	1.5 (0.8, 2.8)	0.189
Diabetes mellitus	7.4 (2.5, 22.1)	<0.001 *
PAD	1.7 (0.9, 3.4)	0.130
CKD	1.1 (0.5, 2.1)	0.883
Age	1.0 (1.0, 1.1)	0.028 *
Reconstruction type		
Local flap	1 [Reference]	-
Free tissue transfer	0.4 (0.2, 0.8)	0.009 *

* Values in bold indicate statistical significance. Abbreviations: OR, odds ratio; CI, confidence interval; MAC, medial arterial calcification; PAD, peripheral arterial disease; CKD, chronic kidney disease.

**Table 11:** Multivariate model for unplanned return to the operating room.

Co-Variate	OR (95% CI)	*p*-Value
MAC	1.6 (0.9, 2.6)	0.087
Diabetes mellitus	1.9 (1.2, 3.0)	0.011 *
PAD	1.7 (1.1, 2.6)	0.023 *
CKD	0.9 (0.5, 1.5)	0.627
Age	1.0 (1.0, 1.0)	**0.131**
Reconstruction type		
Local flap	1 [Reference]	-
Free tissue transfer	1.1 (0.1, 1.9)	0.329

* Values in bold indicate statistical significance. Abbreviations: OR, odds ratio; CI, confidence interval; MAC, medial arterial calcification; PAD, peripheral arterial disease; CKD, chronic kidney disease.

## Data Availability

The datasets presented in this article are not readily available because of HIPAA concerns.

## References

[1] Allison M.A., Armstrong D.G., Goodney P.P., Hamburg N.M., Kirksey L., Lancaster K.J., Mena-Hurtado C.I., Misra S., Treat-Jacobson D.J., White Solaru K.T. (2023). Health disparities in peripheral artery disease: A scientific statement from the American Heart Association. Circulation.

[2] Aday A.W., Matsushita K. (2021). Epidemiology of peripheral artery disease and polyvascular disease. Circ. Res..

[3] Heald A.H., Stedman M., Davies M., Livingston M., Alshames R., Lunt M., Rayman G., Gadsby R. (2020). Estimating life years lost to diabetes: Outcomes from analysis of National Diabetes Audit and Office of National Statistics data. Cardiovasc. Endocrinol. Metab..

[4] Valabhji J., Kar P. (2023). Rise in type 2 diabetes shows that prevention is more important than ever. BMJ.

[5] Lovic D., Piperidou A., Zografou I., Grassos H., Pittaras A., Manolis A. (2020). The growing epidemic of diabetes mellitus. Curr. Vasc. Pharmacol..

[6] Haileamlak A. (2018). Chronic kidney disease is on the rise. Ethiop. J. Health Sci..

[7] Ho C.Y., Shanahan C.M. (2016). Medial arterial calcification: An overlooked player in peripheral arterial disease. Arterioscler. Thromb. Vasc. Biol..

[8] Losurdo F., Casini A., Clerici G., Ferraresi R. Medial Artery Calcification: The Silent Killer of the Leg. https://www.acc.org/latest-in-cardiology/articles/2021/07/12/12/31/medial-artery-calcification.

[9] Lanzer P., Hannan F.M., Lanzer J.D., Janzen J., Raggi P., Furniss D., Schuchardt M., Thakker R., Fok P.-W., Saez-Rodriguez J. (2021). Medial arterial calcification: JACC state-of-the-art review. J. Am. Coll. Cardiol..

[10] Lau W.L., Ix J.H. (2023). Clinical Detection, Risk Factors, and Cardiovascular Consequences of Medial Arterial Calcification: A Pattern of Vascular Injury Associated with Aberrant Mineral Metabolism.

[11] Rocha-Singh K.J., Zeller T., Jaff M.R. (2014). Peripheral arterial calcification: Prevalence, mechanism, detection, and clinical implications. Catheter. Cardiovasc. Interv..

[12] Lehto S., Niskanen L., Suhonen M., Rönnemaa T., Laakso M. (1996). Medial artery calcification: A neglected harbinger of cardiovascular complications in non–insulin-dependent diabetes mellitus. Arterioscler. Thromb. Vasc. Biol..

[13] Niskanen L., Siitonen O., Suhonen M., Uusitupa M.I. (1994). Medial artery calcification predicts cardiovascular mortality in patients with NIDDM. Diabetes Care.

[14] London G.M., Guerin A.P., Marchais S.J., Metivier F., Pannier B., Adda H. (2003). Arterial media calcification in end-stage renal disease: Impact on all-cause and cardiovascular mortality. Nephrol. Dial. Transplant..

[15] Schantl A.E., Ivarsson M.E., Leroux J.C. (2019). Investigational pharmacological treatments for vascular calcification. Adv. Ther..

[16] Opdebeeck B., Van den Branden A., Adriaensen S., Orriss I.R., Patel J.J., Geryl H., Zwijsen K., D’Haese P.C., Verhulst A. (2024). β, γ-Methylene-ATP and its metabolite medronic acid affect both arterial media calcification and bone mineralization in non-CKD and CKD rats. JBMR Plus.

[17] Xie Y., Lin T., Jin Y., Berezowitz A.G., Wang X.-L., Lu J., Cai Y., Guzman R.J. (2024). Smooth muscle cell-specific matrix metalloproteinase 3 deletion reduces osteogenic transformation and medial artery calcification. Cardiovasc. Res..

[18] Van den Branden A., Verhulst A., D’Haese P.C., Opdebeeck B. (2022). New therapeutics targeting arterial media calcification: Friend or foe for bone mineralization?. Metabolites.

[19] Herrington D.M., Mao C., Parker S.J., Fu Z., Yu G., Chen L., Venkatraman V., Fu Y., Wang Y., Howard T.D. (2018). Proteomic architecture of human coronary and aortic atherosclerosis. Circulation.

[20] Chen X., Liu L., Palacios G., Gao J., Zhang N., Li G., Lu J., Song T., Zhang Y., Lv H. (2010). Plasma metabolomics reveals biomarkers of the atherosclerosis. J. Sep. Sci..

[21] Williams J.W., Winkels H., Durant C.P., Zaitsev K., Ghosheh Y., Ley K. (2020). Single cell RNA sequencing in atherosclerosis research. Circ. Res..

[22] Chantelau E., Lee K., Jungblut R. (1997). Distal arterial occlusive disease in diabetes is related to medial arterial calcification. Exp. Clin. Endocrinol. Diabetes.

[23] Soor G.S., Vukin I., Leong S.W., Oreopoulos G., Butany J. (2008). Peripheral vascular disease: Who gets it and why? A histomorphological analysis of 261 arterial segments from 58 cases. Pathology.

[24] Narula N., Dannenberg A.J., Olin J.W., Bhatt D.L., Johnson K.W., Nadkarni G., Min J., Torii S., Poojary P., Anand S.S. (2018). Pathology of peripheral artery disease in patients with critical limb ischemia. J. Am. Coll. Cardiol..

[25] Mustapha J.A., Diaz-Sandoval L.J., Saab F. (2017). Infrapopliteal calcification patterns in critical limb ischemia: Diagnostic, pathologic and therapeutic implications in the search for the endovascular holy grail. J. Cardiovasc. Surg..

[26] Li K.R., Huffman S.S., Gupta N.J., Truong B.N., Lava C.X., Rohrich R.N., Atves J.N., Steinberg J.S., Akbari C.M., Youn R.C. (2024). Refining a Multidisciplinary “Vasculo-plastic” Approach to Limb Salvage: An Institutional Review Examining 300 Lower Extremity Free Flaps. Plast. Reconstr. Surg..

[27] Nigam M., Zolper E.G., Sharif-Askary B., Abdou S.A., Charipova K., Bekeny J.C., Fan K.L., Steinberg J.S., Attinger C.E., Evans K.K. (2022). Expanding criteria for limb salvage in comorbid patients with nonhealing wounds: The MedStar Georgetown Protocol and Lessons Learned after 200 Lower Extremity Free Flaps. Plast. Reconstr. Surg..

[28] Ferraresi R., Ucci A., Pizzuto A., Losurdo F., Caminiti M., Minnella D., Casini A., Clerici G., Montero-Baker M., Mills J. (2021). A novel scoring system for small artery disease and medial arterial calcification is strongly associated with major adverse limb events in patients with chronic limb-threatening ischemia. J. Endovasc. Ther..

[29] Charlson M.E., Pompei P., Ales K.L., MacKenzie C.R. (1987). A new method of classifying prognostic comorbidity in longitudinal studies: Development and validation. J. Chronic Dis..

[30] McHugh M.L. (2012). Interrater reliability: The kappa statistic. Biochem. Med..

[31] Attinger C.E., Ducic I., Cooper P., Zelen C.M. (2002). The role of intrinsic muscle flaps of the foot for bone coverage in foot and ankle defects in diabetic and nondiabetic patients. Plast. Reconstr. Surg..

[32] Berger L.E., Spoer D.L., Huffman S.S., Garrett R.W., Khayat E., DiBello J.R., Zolper E.G., Akbari C.M., Evans K.K., Attinger C.E. (2025). The role of local flaps in foot and ankle reconstruction: An assessment of outcomes across 206 patients with chronic wounds. Plast. Reconstr. Surg..

[33] Rohrich R.N., Li K.R., Lava C.X., Akbari C.M., Attinger C.E. (2024). Angiosome-Guided Revascularization in Local Flap Reconstruction of the Foot and Ankle: Comparable Outcomes With Both Direct and Indirect Revascularization. Ann. Plast. Surg..

[34] Li K.R., Rohrich R.N., Lava C.X., Akbari C.M., Attinger C.E. (2024). Optimizing Lower Extremity Local Flap Reconstruction in Peripheral Vascular Disease. Ann. Plast. Surg..

[35] Li K.R., Lava C.X., Lee S.Y., Suh J., Berger L.E., Attinger C.E. (2024). Optimizing the Use of Pedicled versus Random Pattern Local Flaps in the Foot and Ankle. Plast. Reconstr. Surg. Glob. Open.

[36] Li K.R., Lava C.X., Neughebauer M.B., Rohrich R.N., Atves J., Steinberg J., Akbari C.M., Youn R.C., Attinger C.E., Evans K.K. (2024). A Multidisciplinary Approach to End-Stage Limb Salvage in the Highly Comorbid Atraumatic Population: An Observational Study. J. Clin. Med..

[37] Li K.R., Rohrich R.N., Lava C.X., Gupta N.J., Hidalgo C.M., Episalla N.C., Akbari C.M., Evans K.K. (2024). A Combined “Vasculoplastic” Approach to the Vasculopathic Patient Undergoing Limb Salvage: Understanding the Role of Endovascular Revascularization for Lower Extremity Free Tissue Transfer. J. Reconstr. Microsurg..

[38] Janhofer D.E., Lakhiani C., Kim P.J., Akbari C., Naz I., Tefera E.A., Attinger C.E., Evans K.K. (2019). The utility of preoperative arteriography for free flap planning in patients with chronic lower extremity wounds. Plast. Reconstr. Surg..

[39] Wilson J.A., Clark J.J. (2003). Obesity: Impediment to wound healing. Crit. Care Nurs. Q..

[40] Pierpont Y.N., Dinh T.P., Salas R.E., Johnson E.L., Wright T.G., Robson M.C., Payne W.G. (2014). Obesity and surgical wound healing: A current review. Int. Sch. Res. Not..

[41] Forsythe R., Brownrigg J., Hinchliffe R. (2015). Peripheral arterial disease and revascularization of the diabetic foot. Diabetes Obes. Metab..

[42] Nawaz S., Chinnadurai R., Al-Chalabi S., Evans P., Kalra P.A., Syed A.A., Sinha S. (2023). Obesity and chronic kidney disease: A current review. Obes. Sci. Pract..

[43] Liao X., Shi K., Zhang Y., Huang X., Wang N., Zhang L., Zhao X. (2023). Contribution of CKD to mortality in middle-aged and elderly people with diabetes: The China Health and Retirement Longitudinal Study. Diabetol. Metab. Syndr..

[44] Hacker K. (2024). The Burden of Chronic Disease. Mayo Clin. Proc. Innov. Qual. Outcomes.

[45] Sundaram S., Barksdale C., Rodriguez S., Wooster M.D. (2025). The Impact of Small Artery Disease (SAD) and Medial Arterial Calcification (MAC) Scores on Chronic Wound and Amputation Healing: Can It Tell Us More?. Ann. Vasc. Surg..

[46] Moawad M.R. (2022). Management of Peripheral Arterial Calcification. Cardiovascular Calcification.

[47] Sage A.P., Tintut Y., Demer L.L. (2010). Regulatory mechanisms in vascular calcification. Nat. Rev. Cardiol..

[48] Nikolajević J., Šabovič M. (2023). Inflammatory, metabolic, and coagulation effects on medial arterial calcification in patients with peripheral arterial disease. Int. J. Mol. Sci..

[49] Bonomini F., Rodella L.F., Rezzani R. (2015). Metabolic syndrome, aging and involvement of oxidative stress. Aging Dis..

[50] Mozaffarian D., Kamineni A., Prineas R.J., Siscovick D.S. (2008). Metabolic syndrome and mortality in older adults: The Cardiovascular Health Study. Arch. Intern. Med..

[51] Malik S., Wong N.D., Franklin S.S., Kamath T.V., L’Italien G.J., Pio J.R., Williams G.R. (2004). Impact of the metabolic syndrome on mortality from coronary heart disease, cardiovascular disease, and all causes in United States adults. Circulation.

[52] Krakauer N.Y., Krakauer J.C. (2018). Anthropometrics, metabolic syndrome, and mortality hazard. J. Obes..

[53] Ford E.S. (2005). Risks for all-cause mortality, cardiovascular disease, and diabetes associated with the metabolic syndrome: A summary of the evidence. Diabetes Care.

[54] Chopra A., Azarbal A.F., Jung E., Abraham C.Z., Liem T.K., Landry G.J., Moneta G.L., Mitchell E.L. (2018). Ambulation and functional outcome after major lower extremity amputation. J. Vasc. Surg..

[55] Attinger C.E., Brown B.J. (2012). Amputation and ambulation in diabetic patients: Function is the goal. Diabetes/Metab. Res. Rev..

